# Revisiting False-Positive and Imitated Dissociative Identity Disorder

**DOI:** 10.3389/fpsyg.2021.637929

**Published:** 2021-05-06

**Authors:** Igor Jacob Pietkiewicz, Anna Bańbura-Nowak, Radosław Tomalski, Suzette Boon

**Affiliations:** Research Centre for Trauma & Dissociation, SWPS University of Social Sciences and Humanities, Katowice, Poland

**Keywords:** dissociative identity disorder (DID), false-positive cases, personality disorder, dissociation, differential diagnosis

## Abstract

ICD-10 and DSM-5 do not provide clear diagnosing guidelines for DID, making it difficult to distinguish ‘genuine’ DID from imitated or false-positive cases. This study explores meaning which patients with false-positive or imitated DID attributed to their diagnosis. 85 people who reported elevated levels of dissociative symptoms in SDQ-20 participated in clinical assessment using the Trauma and Dissociation Symptoms Interview, followed by a psychiatric interview. The recordings of six women, whose earlier DID diagnosis was disconfirmed, were transcribed and subjected to interpretative phenomenological analysis. Five main themes were identified: (1) endorsement and identification with the diagnosis. (2) The notion of dissociative parts justifies identity confusion and conflicting ego-states. (3) Gaining knowledge about DID affects the clinical presentation. (4) Fragmented personality becomes an important discussion topic with others. (5) Ruling out DID leads to disappointment or anger. To avoid misdiagnoses, clinicians should receive more systematic training in the assessment of dissociative disorders, enabling them to better understand subtle differences in the quality of symptoms and how dissociative and non-dissociative patients report them. This would lead to a better understanding of how patients with and without a dissociative disorder report core dissociative symptoms. Some guidelines for a differential diagnosis are provided.

## Introduction

Multiple Personality Disorder (MPD) was first introduced in DSM-III in 1980 and re-named Dissociative Identity Disorder (DID) in subsequent editions of the diagnostic manual (American Psychiatric Association, 2013). Table 1 shows diagnostic criteria of this disorder in ICD-10, ICD-11, and DSM-5. Some healthcare providers perceive it as fairly uncommon or associated with temporary trends (Brand et al., 2016). Even its description in ICD-10 (World Health Organization, 1993) starts with: “This disorder is rare, and controversy exists about the extent to which it is iatrogenic or culture-specific” (p. 160). Yet, according to the guidelines of the International Society for the Study of Trauma and Dissociation (International Society for the Study of Trauma and Dissociation, 2011), the prevalence of DID in the general population is estimated between 1 and 3%. The review of global studies on DID in clinical settings by Sar (2011) shows the rate from 0.4 to 14%. However, in studies using clinical diagnostic interviews among psychiatric in-patients, and in European studies these numbers were lower (Friedl et al., 2000). The discrepancies apparently depend on the sample, the methodology and diagnostic interviews used by researchers.

**TABLE 1:** Diagnostic criteria for dissociative identity disorder.

**ICD-10 Multiple personality disorder F44.81** (A) Two or more distinct personalities exist within the individual, only one being evident at a time. (B) Each personality has its own memories, preferences, and behavior patterns, and at some time (and recurrently) takes full control of the individual’s behavior. (C) There is inability to recall important personal information which is too extensive to be explained by ordinary forgetfulness. (D) The symptoms are not due to organic mental disorders (F00–F09) (e.g., in epileptic disorders) or to psychoactive substance-related disorders (F10–F19) (e.g., intoxication or withdrawal).
**ICD-11 Dissociative identity disorder 6B64** Dissociative identity disorder is characterized by disruption of identity in which there are two or more distinct personality states (dissociative identities) associated with marked discontinuities in the sense of self and agency. Each personality state includes its own pattern of experiencing, perceiving, conceiving, and relating to self, the body, and the environment. At least two distinct personality states recurrently take executive control of the individual’s consciousness and functioning in interacting with others or with the environment, such as in the performance of specific aspects of daily life such as parenting, or work, or in response to specific situations (e.g., those that are perceived as threatening). Changes in personality state are accompanied by related alterations in sensation, perception, affect, cognition, memory, motor control, and behavior. There are typically episodes of amnesia, which may be severe. The symptoms are not better explained by another mental, behavioral or neurodevelopmental disorder and are not due to the direct effects of a substance or medication on the central nervous system, including withdrawal effects, and are not due to a disease of the nervous system or a sleep-wake disorder. The symptoms result in significant impairment in personal, family, social, educational, occupational, or other important areas of functioning.
**DSM-5 Dissociative identity disorder 300.14** (A) Disruption of identity characterized by two or more distinct personality states, which may be described in some cultures as an experience of possession. The disruption in identity involves marked discontinuity in sense of self and sense of agency accompanied by related alterations in affect, behavior, consciousness, memory, perception, cognition, and/or sensory-motor functioning. These signs and symptoms may be observed by others or reported by the individual. (B) Recurrent gaps in the recall of everyday events, important personal information, and/or traumatic events that are inconsistent with ordinary forgetting. (C) The symptoms cause clinically significant distress or impairment in social, occupational, or other important areas of functioning. (D) The disturbance is not a normal part of a broadly accepted cultural or religious practice. *Note:* In children, the symptoms are not better explained by imaginary playmates or other fantasy play. (E) The symptoms are not attributable to the physiological effects of a substance (e.g., blackouts or chaotic behavior during alcohol intoxication) or another medical condition (e.g., complex partial seizures).

Diagnosing complex dissociative disorders (DID or Other Specified Dissociative Disorder, OSDD) is challenging for several reasons. Firstly, patients present a lot of avoidance and rarely report dissociative symptoms spontaneously without direct questioning (Boon and Draijer, 1993; International Society for the Study of Trauma and Dissociation, 2011; Dorahy et al., 2014). In addition, standard mental state examination does not include these symptoms and healthcare professionals do not receive appropriate training in diagnosing dissociative disorders (Leonard et al., 2005). Secondly, complex dissociative disorders are polysymptomatic, and specialists would rather diagnose these patients with disorders more familiar to them from clinical practice, e.g., anxiety disorders, eating disorders, schizophrenia, or borderline personality disorder (Boon and Draijer, 1995; Dell, 2006; Brand et al., 2016). For these reasons, complex dissociative disorders are underdiagnosed and often mis-diagnosed. For example, 26.5–40.8% of DID patients would already have been diagnosed and treated for schizophrenia (Putnam et al., 1986; Ross et al., 1989). On the other hand, because there is so much information about DID in the media (Hollywood productions, interviews and testimonies published on YouTube, blogs), people who are confused about themselves and try to find an accurate diagnosis for themselves may learn about DID symptoms on the Internet, identify themselves with the disorder, and later (even unintentionally) report core symptoms in a very convincing way (Draijer and Boon, 1999). This presents a risk of making a false positive diagnosis, which is unfavorable for the patient, because using treatment developed for DID with patients without autonomous dissociative parts may be inefficient or even reinforce their pathology.

Authors who wrote about patients inappropriately diagnosed with this disorder used terms such as ‘malingering’ or ‘factitious’ DID (Coons and Milstein, 1994; Thomas, 2001). According to Draijer and Boon (1999), both labels imply that patients intentionally simulate symptoms, either for external gains (financial benefits or justification for one’s actions in court) or for other forms of gratification (e.g., interest from others), while in many cases their motivation is not fully conscious. Getting a DID diagnosis can also provide structure for inner chaos and incomprehensible experiences, and be associated with hope and belief it is real. On the other hand, diagnostic errors often result in inappropriate treatment plans and procedures.

Already in 1995 Boon and Draijer stressed that a growing number of people self-diagnosed themselves based on information from literature and the Internet, and reported symptoms by the book during psychiatric or psychological assessment. Based on their observation of 36 patients in whom DID had been ruled out after applying the structured clinical interview SCID-D, these clinicians identified differences between genuine and imitated DID. They classified their participants into three groups: (1) borderline personality disorder, (2) histrionic personality disorder, or (3) persons with severe dissociative symptoms but not DID. Participants in that study reported symptoms similar to DID patients, including: amnesia (but only for unacceptable behavior), depersonalisation, derealisation, identity confusion, and identity alteration. However, they presented themselves and interacted with the therapist in very different ways. While DID patients are usually reluctant to talk about their symptoms and experience their intrusions as shameful, people who imitated DID were eager to present their problems, sometimes in an exaggerated way, in an attempt to convince the clinician that they suffered from DID (Boon and Draijer, 1995; Draijer and Boon, 1999). Similar observations were expressed by Thomas (2001) saying that people with imitated DID can present their history chronologically, using the first person even when they are highly distressed or allegedly presenting an altered personality, and are comfortable with disclosing information about experiences of abuse. They can talk about intrusions of dissociative parts, hearing voices or difficulties controlling emotions, without shame.

Unfortunately, ICD-10, ICD-11, and DSM-5 offer no specific guidelines on how to differentiate patients with personality disorders and dissociative disorders by the manner in which they report symptoms. There are also limited instruments to distinguish between false-positive and false-negative DID. From the clinical perspective, it is also crucial to understand the motives for being diagnosed with DID, and disappointment when this diagnosis is disconfirmed. Accurate assessment can contribute to developing appropriate psychotherapeutic procedures (Boon and Draijer, 1995; Draijer and Boon, 1999). Apart from observations already referred to earlier in this article, there are no qualitative analyses of false-positive DID cases in the past 20 years. Most research was quantitative and compared DID patients and simulators in terms of cognitive functions (Boysen and VanBergen, 2014). This interpretative phenomenological analysis is an idiographic study which explores personal experiences and meaning attributed to conflicting emotions and behaviors in six women who had previously been diagnosed with DID and referred to the Research Centre for Trauma and Dissociation for re-evaluation. It explores how they came to believe they have DID and what had led clinicians to assume that these patients could be suffering from this disorder.

## Materials and Methods

This study was carried out in Poland in 2018 and 2019. Rich qualitative material collected during in-depth clinical assessments was subjected to the interpretative phenomenological analysis (IPA), a popular methodological framework in psychology for exploring people’s personal experiences and interpretations of phenomena (Smith and Osborn, 2008). IPA was selected to build a deeper understanding of how patients who endorsed and identified with dissociative identity disorder made sense of the diagnosis and what it meant for them to be classified as false-positive cases during reassessment.

Interpretative phenomenological analysis uses phenomenological, hermeneutic, and idiographic principles. It employs ‘double hermeneutics,’ in which participants share their experiences and interpretations, followed by researchers trying to make sense and comment on these interpretations. IPA uses small, homogenous, purposefully selected samples, and data are carefully analyzed case-by-case (Smith and Osborn, 2008; Pietkiewicz and Smith, 2014).

### Procedure

This study is part of a larger project examining alterations in consciousness and dissociative symptoms in clinical and non-clinical groups, held at the Research Centre for Trauma & Dissociation, financed by the National Science Centre, and approved by the Ethical Review Board at the SWPS University of Social Sciences & Humanities. Potential candidates enrolled themselves or were registered by healthcare providers via an application integrated with the website www.e-psyche.eu. They filled in demographic information and completed online tests, including: Somatoform Dissociation Questionnaire (SDQ-20, Pietkiewicz et al., 2018) and Trauma Experiences Checklist (Nijenhuis et al., 2002). Those with elevated SDQ-20 scores (above 28 points) or those referred for differential diagnosis were consulted and if dissociative symptoms were confirmed, they were invited to participate in an in-depth clinical assessment including a series of interviews, video-recorded and performed at the researcher’s office by the first author who is a psychotherapist and supervisor experienced in the dissociation field. In Poland, there are no gold standards for diagnosing dissociative disorders. The first interview was semi-structured, open-ended and explored the patient’s history, main complaints and motives for participation. It included questions such as: What made you participate in this study? What are your main difficulties or symptoms in daily life? What do you think caused them? Further questions were then asked to explore participants’ experiences and meaning-making. This was followed by the Trauma and Dissociation Symptoms Interview (TADS-I, Boon and Matthess, 2017). The TADS-I is a new semi-structured interview intended to identify DSM-5 and ICD-11 dissociative disorders. The TADS-I differs in several ways from other semi-structured interviews for the assessment of dissociative disorders. Firstly, it includes a significant section on somatoform dissociative symptoms. Secondly, it includes a section addressing other trauma-related symptoms for several reasons: (1) to obtain a more comprehensive clinical picture of possible comorbidities, including symptoms of PTSD and complex PTSD, (2) to gain a better insight into the (possible) dissociative organization of the personality: patient’s dissociative parts hold many of these comorbid symptoms and amnesia, voices or depersonalisation experiences are often associated with these symptoms; and (3) to better distinguish between complex dissociative disorders, personality disorders and other Axis I disorders and false positive DID. Finally, the TADS-I also aims to distinguish between symptoms of pathological dissociation indicating a division of the personality and symptoms which are related to a narrowing or a lowering of consciousness, and not to the structural dissociation of the personality. Validation testing of the TADS-I is currently underway. TADS interviews ranging from 2 to 4 h were usually held in sessions of 90 min. Interview recordings were assessed by three healthcare professionals experienced in the dissociation field, who discussed each case and consensually came up with a diagnosis based on ICD-10. An additional mental state examination was performed by the third author who is a psychiatrist, also experienced in the differential diagnosis of dissociative disorders. He collected medical data, double-checked the most important symptoms, communicated the results and discussed treatment indications. Qualitative data collected from six patients out of 85 were selected for this interpretative phenomenological analysis, based on the following criteria for inclusion, which could ensure a homogenous sample expected of IPA studies – (a) female, (b) previously diagnosed or referred to rule in/out DID, (c) endorsement and identification with DID, (d) dissociative disorder disconfirmed in the assessment. Interviews with every participant in this study ranged from 3 h 15 min to 7 h 20 min (mean: 6 h).

### Participants

Participants of this IPA were six female patients aged between 22 and 42 years who were selected out of 86 people examined in a larger study exploring dissociation and alterations in consciousness in clinical and non-clinical groups. (Participants in the larger study met criteria of different diagnoses and seven among them had ‘genuine’ DID). These six patients did not meet DID criteria on the TADS-I interview but believed themselves that they qualified for that diagnosis. Four of them had higher education, two were secondary school graduates. All of them registered in the study by themselves hoping to confirm their diagnosis but two (Olga and Katia) were referred by psychiatrists, and the others by psychotherapists. All of them traveled from far away, which showed their strong motivation to participate in the assessment. Four had previously had psychiatric treatment and five had been in psychotherapy due to problems with emotional regulation and relationships. In the cases of Victoria and Dominique, psychotherapy involved working with dissociative parts. None of them recalled any physical or sexual abuse, but three (Dominique, Victoria, and Mary), following therapists’ suggestions, were trying to seek such traumatic memories to justify their diagnosis. They all felt emotionally neglected by carriers in childhood and emotionally abused by significant others. None of them reported symptoms indicating the existence of autonomous dissociative parts. None had symptoms indicating amnesia for daily events, but four declared not remembering single situations associated with conflicting emotions, shame, guilt, or conversations during which they were more focused on internal experiences rather than their interlocutors. None experienced PTSD symptoms (e.g., intrusive traumatic memories and avoidance), autoscopic phenomena (e.g., out-of-body experiences), or clinically significant somatoform symptoms. None had auditory verbal hallucinations but four intensely engaged in daydreaming and experienced imagined conversations as very real. All of them had been seeking information about DID in literature and the Internet. For more information about them see Table 2. Their names have been changed to protect their confidentiality.

**TABLE 2:** Study participants.

Name	Participant’s characteristics
Victoria	Age 22, single, lives with parents and younger brother. Stopped her studies after 3 years and was hospitalized in a psychiatric facility for a short period due to problems with emotions and relationships. Reports difficulties with recognizing and expressing emotions, emptiness, feels easily hurt and rejected, afraid of abandonment. Perceives herself as unimportant and worthless, sometimes cuts herself for emotional relief. Maintains superficial relationships, does not trust people; in childhood was frequently left alone with grandparents because her parents traveed; described her parents as setting high expectations, mother as getting easily upset and impulsive. No substance use. No history of physical or sexual trauma. Her maternal grandfather abused alcohol but was not violent; no history of suicides in her family. Scored 38 points in SDQ-20 but no significant somatoform symptoms reported during clinical assessment.
Karina	Age 22, single, secondary education. Enrolled in university programs twice but stopped. Acting is a hobby; recently worked as a waitress or hostess, currently unemployed. Has had psychiatric treatment for 17 years due to anxiety and problems in relationships. Two short hospital admissions; in psychodynamic psychotherapy in last 2 years. Reports emotional instability, feeling depressed, anxious, and lonely; maintains few relationships; experiences conflicts with expressing anger and needs for dependency, no self-harm. She had periods of using alcohol excessively in the past, currently once a month, no drugs. No family members used psychiatric help. Reports abandonment, emotional and physical abuse in childhood and eagerly talks about these experiences. Scored 68 points in SDQ-20 but no significant somatoform symptoms reported during clinical assessment.
Dominique	Age 33, higher education, married, three children. Works as a playwright, comes from an artistic family. Was given away to her grandparents as a baby and returned to parents and brothers when she was seven; often felt abandoned and neglected. She had learning difficulties and problems in relationships, mood regulation, auto-aggressive behavior, feelings of emptiness and loneliness. Denies using alcohol or drugs; at secondary school abused marihuana. Her paternal grandmother had psychosis, her father abused marihuana and mother was treated for depression. Reports poverty at home. No suicides in family. Often retreated into her fantasy world in which she developed a story about boys kept in a resocialisation center. Has had psychiatric treatment and counseling for 20 years. Scored 52 points in SDQ-20 but no somatoform symptoms confirmed during clinical assessment.
Mary	Age 34, higher education, married. Works in the creative industry and engaged in proselytic activities as an active Jehovah’s Witness (joined the organization 10 years earlier, encouraged by her mother). Has had EMDR therapy for 2 years due to problems maintaining relationships and managing anger. When her therapist asked if she felt there were different parts inside her, she started exploring information about DID. She denies smoking or using any drugs, alcohol. Mother suffered from mild depression. No suicides in family. Scored 48 points in SDQ-20 but no somatoform symptoms confirmed during clinical assessment.
Olga	Age 40, higher education, single. Works in social care. Reports depressive mood, low self-esteem, difficulties with concentration, problems with social contacts. Occasionally uses alcohol in small doses, no drugs. Describes her mother as demanding but also distant and negligent because she was busy with her medical practice. Father withdrawn and depressed but never used psychiatric treatment. No other trauma history. No suicides in family. Tried psychotherapy four times but usually terminated treatment after a while. Her psychiatrist referred her for evaluation of memory problems, and confirming DID. Scored 31 points in SDQ-20; confirms a few somatoform symptoms: headaches, symptoms associated with cystitis, detachment from bodily sensations.
Katia	Age 42, post-graduate education. Unemployed. On social benefits for 15 years due to neurological and pulmonary symptoms, complications after urological surgeries. Reports low self-esteem, self-loathing, problems in establishing or maintaining relationships, feeling lonely, rejected and not understood. Inclinations toward passive-aggressive behavior toward people representing authority, fatigue, insecurity about her financial situation. Reports no alcohol or drug use. Mother treated for depression. No suicides in family. Scored 69 points in SDQ-20; multiple somatic complaints associated with Lyme disease, describes mother as emotionally and physically abusive, and father as abandoning and unprotecting. Has never used psychotherapy; was referred for consultation by a psychiatrist after persuading him that she had DID symptoms.

*Participants names have been changed to protect their confidentiality.*

### The Researchers

The principal investigator (IJP) is a psychotherapist, supervisor, and researcher in the field of community health psychology and clinical psychology. The second co-investigator (RT) is a psychiatrist, psychotherapist, and supervisor. The third co-investigator (SB) is a clinical psychologist, psychotherapist, supervisor, and a consulting expert in forensic psychology, who also developed the TADS-I. They are all mentors and trainers of the European Society for Trauma and Dissociation, with significant expertise in the assessment of post-traumatic conditions. The first co-investigator (AB) has a master’s degree in psychology and is a Ph.D. candidate. She is also a psychotherapist in training. All authors coded and discussed their understanding of data. Their understanding and interpretations of symptoms reported by participants were influenced by their background knowledge and experience in diagnosing and treating patients with personality disorders and dissociative disorders.

### Data Analysis

Verbatim transcriptions were made of all video recordings, which were analyzed together with researchers’ notes using qualitative data-analysis software – NVivo11. Consecutive analytical steps recommended for IPA were employed in the study (Pietkiewicz and Smith, 2014). For each interview, researchers watched the recording and carefully read the transcript several times. They individually made notes about body language, facial expressions, the content and language use, and wrote down their interpretative comments using the ‘annotation’ feature in NVivo10. Next, they categorized their notes into emergent themes by allocating descriptive labels (nodes). The team then compared and discussed their coding and interpretations. They analyzed connections between themes in each interview and between cases, and grouped themes according to conceptual similarities into main themes and sub-themes.

### Credibility Checks

During each interview, participants were encouraged to give examples illustrating reported symptoms or experiences. Clarification questions were asked to negotiate the meaning participants wanted to convey. At the end of the interview, they were also asked questions to check that their responses were thorough. The researchers discussed each case thoroughly and also compared their interpretative notes to compare their understanding of the content and its meaning (the second hermeneutics).

## Results

Participants in this study explained how they concluded they were suffering from DID, developed knowledge about the syndrome and an identity of a DID patient, and how this affected their everyday life and relationships. Five salient themes appeared in all interviews, as listed in Table 3. Each theme is discussed and illustrated with verbatim excerpts from the interviews, in accordance with IPA principles.

**TABLE 3:** Salient themes identified during the interpretative phenomenological analysis.

Theme 1:	Endorsement and identification with the diagnosis
Theme 2:	Using the notion of dissociative parts to justify identity confusion and conflicting ego-states
Theme 3:	Gaining knowledge about DID affects the clinical presentation
Theme 4:	Fragmented personality becomes an important discussion topic with others
Theme 5:	Ruling out DID leads to disappointment or anger.

### Theme 1: Endorsement and Identification With the Diagnosis

All six participants hoped to confirm they had DID. They read books and browsed the Internet seeking information about dissociation, and watched YouTube videos presenting people describing multiple personalities. Dominique, Victoria, Mary, and Karina said that a mental health professional suggested this diagnosis to them. Dominique remembers consulting a psychiatrist when she was 15, because she had problems controlling anger at home or in public places. She initially found descriptions of borderline personality captured her experiences well enough, but a psychiatrist refuted the idea and recommended further diagnostics toward a dissociative disorder. However, the girl refused to go to hospital for observation.

During an argument with my mother I felt as if some incredible force took control and I smashed the glass in the cabinet with my hand. It was like being under control of an alien force. I started reading about borderline and I thought I had it. I found a webpage about that and told my mother I should see a psychiatrist. I went for a consultation and told her my story. This lady said: “Child, you don’t have borderline, but multiple personality.” She wanted to keep me in the psychiatric unit but I did not agree to stay for observation. (Dominique).

This led Dominique to research the new diagnosis. Karina also said she was encouraged to seek information about DID, when a doctor suggested she might be suffering with it.

When I was 11, I had problems at school and home. Other children made fun of me. My mom took me to a doctor and he said I had borderline, but later I was diagnosed with an anxiety disorder. That doctor also suggested I had DID and told me that I should read more about this diagnosis. (Karina).

Victoria and Mary shared similar stories about psychotherapists suggesting the existence of dissociative parts, having readily accepted this new category as a good explanation for aggressive impulses or problems with recalling situations evoking guilt or shame. Dominique and Victoria stressed, however, that, apart from feeling emotionally abandoned, they could not trace any significant traumas in their early childhoods, although therapists maintained that such events must be present in dissociative patients.

I have no idea why I have this [DID]. My therapist looked for evidence of childhood trauma, which sounds like the easiest explanation, but I don’t feel I had any horrific memories which I threw out of my consciousness. (Victoria).

Katia and Olga had used psychiatric treatment for anxiety and depression for years. After exploring information about different mental disorders they concluded they had DID. They thought there was a similarity between their personal experiences and those of people publishing testimonials about multiple personalities.

I tried to understand this battle inside, leading me to stagnation. I didn’t know how to describe that but I recently bought a book *Healing the fragmented selves of trauma survivors*, and everything was explained there. Some of these things I have discovered myself and some were new to me. (Olga).

Subsequently, Katia presented to her doctor a review of literature about DID, trying to persuade him that she had this disorder.

### Theme 2: Using the Notion of Dissociative Parts to Justify Identity Confusion and Conflicting Ego-States

Once participants had embraced the idea of having multiple personalities, they seemed to construct inner reality and justify conflicting needs, impulses or behaviors as an expression of dissociative parts. They referred to being uncertain about who they were and having difficulties recognizing personal emotions, needs or interests. Some of them felt it was connected to a negative cognition about themselves as worthless, unimportant, and not deserving to express what they felt or wanted. Victoria said she would rather define herself through the eyes of others:

My therapist asked what I wanted or needed. It turned out that without other people’s expectations or preferences to which I normally adjust, I wouldn’t know who I am or what I want. I usually engage in my friends’ hobbies and do what I think gives them pleasure. Otherwise, I think they will not like me and reject me, because I have nothing to offer. (Victoria).

Since a young age, Dominique tended to immerse herself in a fantasy world, developing elaborated scenarios about people living in a youth center administered by a vicious boss. Different characters in her ‘Story’ represented specific features, interests and plans she had.

Well, there is John who is a teacher and researcher. He teaches mathematics. I have no skills in maths at all. Tim is a philosopher and would like to train philosophers, enroll doctoral studies. He would like me to study philosophy but the rest of the system wants me to be a worrier. Ralf is a caring nurse and would like to become a paramedic. It is difficult to reconcile all these different expectations. Whoever comes up front, then I have these ideas. (Dominique).

Dominique neither had amnesia nor found evidence for leading separate lives and engaging herself in activities associated with her characters. She maintained her job as a playwright, and merely imagined alternative scenarios of her life, expressed by her inner heroes. In other parts of the interview, she referred to them as ‘voices inside,’ but admitted she never heard them acoustically. They were her own vivid thoughts representing different, conflicting opinions or impulses.

Katia said she felt internally fragmented. There were times when she engaged in certain interests, knowledge and skills, but she later changed her goals. Fifteen years ago she gave up her academic career and went on sickness benefit when she became disabled due to medical problems; she experienced this as a great loss, a failure, which affected her sense of identity and purpose.

In recent years I have a growing sense of identity fragmentation. I have problems with defining my identity because it changes. I used to feel more stable in the past. I had these versions of myself which were more dominating, so I had a stronger sense of identity. For example, 20 years ago there was this scientist. I was studying and felt like a scientist, attending conferences. Now I don’t have that and I don’t know who I am. […] I also have changing interests and hobbies because of different personalities. Long ago I liked certain music, played the guitar, sang songs. I don’t do that anymore, I suddenly lost interest in all that. (Katia).

She described changes in her professional and social lives in terms of switches between dissociative parts. Although she maintained the first person narrative (“I was studying,” “I played,” or “I sang”), indicating some sense of continuity, she thought it proved the existence of two or more distinct personalities.

Participants also reported thoughts, temptations, impulses or actions which seemed to evoke conflicting feelings. Attributing them to ‘something inside that is not-me’ could free them from guilt or shame, so they used a metaphor of someone taking over, logging in, or switching. Dominique thought it was inappropriate to express disappointment or anger, but she accepted the thought that her dissociative parts were doing this.

When I’m angry at my therapist, it is not really me but somebody inside who gets angry easily. Greg often switches on in such situations and says: “Tell her this and this”. […] I went to a shop once and discovered that the price on the label was not for a whole package of batteries but a single one. And suddenly Greg switched on and had a row with the cashier. I mean, I did it, but wound up by his anger. This is so weird, I wouldn’t react like that. They just charged incorrectly and I would normally ignore that but Greg said: “I give a shit about their mistakes. I won’t accept that.” What a failure! (Dominique).

Mary said she had parts that expressed anger, sadness, and needs associated with attachment. She observed them and allowed them to step in, when situations required.

There were situations in my life when the teenager must have been active. She protected me. She is ready to fight; I am not like that at all. I hate violence, and that teenager likes using force to protect me. […] My therapist suggested I call her after this interview if I do not feel well. I didn’t accept that but the [inner] girls got upset and told me I needed her help. They made me comply, so I agreed to call her if I do not feel well. It has always been like this. (Mary).

During assessment, no participant provided evidence for the existence of autonomous dissociative parts. It seems that the inner characters described by them personified unintegrated ego-states which used to evoke conflicting feelings.

### Theme 3: Exploring Personal Experiences via the Lens of Dissociation

Reading books, websites and watching videos of people who claimed to have DID, encouraged them to compare themselves, talk about and express ‘multiple personalities.’ The participants became familiar with specialist terms and learned about core symptoms mentioned in psychiatric manuals.

I read *First person plural* which helped me understand what this is all about. *The drama of the gifted child* and *The body keeps the score*. More and more girls started to appear. There is a 6-month old baby which showed up only 2 months ago, a sad 11-year old teenager, and a 16-year old who thinks I am a loser. I was a teenager like that. Now she is having problems and becoming withdrawn there are fewer switches, because she knows we need to help the little one first. (Mary).

Olga was also inspired by books. Not only did she find similarities to trauma survivors but she made new discoveries and thought there were other experiences she had been unaware of earlier. Victoria started using techniques which literature recommended for stabilization in dissociative disorders. She said these books helped her understand intense emotions and improve concentration.

This explains everything that happens to me, why I get so angry. I also found anchors helpful. I focus on certain objects, sounds or smells which remind me where I am, instead of drifting away into my thoughts. (Victoria).

It seemed that exploring information about DID encouraged changes in participants’ clinical presentation. At first, they merely struggled with emotional liability or detachment, internal conflicts, and concentration problems. Later, they started reporting intrusions of dissociative parts or using clinical terms (e.g., flashback) for experiences which were not necessarily clinical symptoms. Dominique said that the characters of her story would often ‘log in’ and take control. She demonstrated that during the interview by changing her voice and going into a ‘trance.’ She created her own metaphors, explaining these experiences and comparing them with those described in literature. She stressed that she never had amnesia and remained aware of what was happening during her ‘trance.’

I think it is a form of dissociation on the emotional level. I read a lot… *The minds of Billy Milligan* or *First person plural*. For sure, I do not have an alteration of personality. I have co-consciousness. My theory is, we are like a glove, we all stem from one trunk, but we are like separate fingers. (Dominique).

While participants maintained they had flashbacks, they understood them as sudden recollections of past memories but not necessarily related to trauma. Katia said she recently remembered the picture of the house and garden where she played as a child and associated these experiences with moments of joy. Karina also exemplified her flashbacks with ‘intrusions of happy memories’ which belonged to other personalities:

Sometimes I begin to laugh but this is not my laughter, but the laughter of sheer joy. Someone inside me is very happy and wants to talk about happy childhood memories, make jokes. (Karina).

Mary said a child part of her was responsible for flashbacks and making comments about current situations. However, she later denied hearing voices or having any other Schneider’s symptoms.

I can hear her comments, that she does not like something. I can be flooded by emotions and have flashbacks associated with that child. For example, there is a trigger and I can see things that this child has seen. She is showing me what was happening in her life. (Mary).

Participants discussed their dissociative parts, their names and features, exhibiting neither avoidance nor fear or shame. On the contrary, they seemed to draw pleasure by smiling, showing excitement and eagerness to produce more examples of their unusual experiences. At the beginning of the interview, Karina was very enthusiastic and said, “My heart is beating so fast, as if I were in fight-or-flight mode.”

### Theme 4: Talking About DID Attracts Attention

Not only were multiple personalities a helpful metaphor for expressing conflicting feelings or needs (already mentioned in Theme 2), but they also became an important topic of conversations with family or friends.

My husband says sometimes: “I would like to talk to the little girl.” He then says that I start behaving differently. I also talk to my therapist using different voices. Sometimes, she addresses them asking questions. If questions are asked directly, they respond, but there are times I do not allow them to speak, because the teenager part can be very mean and attacks people. (Mary).

It may have been easier for Mary to express her needs for dependency and care by ascribing them to a little girl and, because she felt awkward about feeling angry with the therapist, attributing hostile impulses to a teenager could give her a sense of control and reduce guilt. Karina decided to create a video-blog for documenting dissociative parts, and shared her videos with people interested in DID. She said she was surprised to find clips in which she looked dreadful, having her make-up smeared all over the face, because she had no memory of doing that. However, she showed no signs that it bothered her. She discussed the videos with her best friend, a DID fan who had encouraged her to enroll in the study in order to confirm her diagnosis. They were collecting evidence to support the idea that she had a dissociative disorder, which she presented one by one, before being asked about details.

Mark [her friend] reads a lot about DID. He says I sometimes talk in a high voice which is not the way I usually talk. He refers to us as plural. […] In some of these videos I do not move or blink for a minute. I look at some point and there is no expression on my face. I can remember things until this moment, and later I discover myself looking like something from Creepypastas. I am so sorry for people who have to see this… and I found my diary. I have been writing diaries since I was seven. I sometimes have no memory for having written something. I need to find these notes because I would like to write a book about a fantasy world and inner conflicts. (Karina).

Dominique and Katia also wrote journals to record dissociative experiences. Katia hoped to be recognized as an expert-by-experience and develop her career in relation to that. She brought with her a script of a book she hoped to publish 1 day.

### Theme 5: Ruling Out DID Leads to Disappointment or Anger

Four participants were openly disappointed that their DID diagnosis was not confirmed. They doubted if their descriptions were accurate enough, or they challenged the interviewer’s understanding of the symptoms. Katia also suggested that she was incapable of providing appropriate answers supporting her diagnosis due to amnesia and personality alterations.

Do you even consider that I might give different answers if you had asked these questions 2 or 5 years ago? I must have erased some examples from my memory and not all experiences belong to me. I know that people can unconsciously modify their narratives and that is why I wanted an objective assessment. […] Nobody believed I was resistant to anesthetics until I was diagnosed with some abnormalities. It was once written in my medical report that I was a hypochondriac. One signature and things become clear to everyone. Sometimes it is better to have the worst diagnosis, but have it. (Katia).

She expected that the diagnosis would legitimize her inability to establish satisfactory relationships, work, and become financially independent. For this reason, she also insisted that the final report produced for her should contain information about how she felt maltreated by family or doctors, and revealed her hopes to claim damages for health injury. Mary and Karina were also upset that the interviewers did not believe they had DID.

Can you try to imagine how hard it is? I am not making things up? You don’t believe me. I am telling you things and you must be thinking, from the adult perspective: “You are making this up.” Nothing pisses me off more than someone who is trying to prove to others that they have just imagined things. They [dissociative parts] feel neglected again, as always! (Mary).

Karina tried to hide her disappointment and claimed she was glad she didn’t have a severe mental illness. However, she thought she would need to build another theory explaining her symptoms. After the interview, she sent more videos trying to prove the assessment results were not accurate.

What about my problems then? I am unable to set boundaries, I have anxiety, I fear that a war might break out. If this is not dissociation, then what? I had tests and they ruled out any neurological problems. I came here and ruled out another possibility. It is some information but I have not heard anything new. (Karina).

Only Victoria seemed relieved that her DID diagnosis was not confirmed. She was happy to discuss how attachment problems or conflicts with expressing emotions and needs affected her social life and career, and receive guidelines for future treatment. She felt liberated from having to uncover childhood traumas that her therapist expected her to have as a dissociative patient.

I was hoping that you would find another explanation for my problems… for what is wrong with me, why I feel so sensitive or spaced out, because it is annoying. I would like to know what is going on. I don’t think I’ve had any severe trauma but everybody wants to talk about trauma all the time. (Victoria).

## Discussion

ICD-10 and DSM-5 provide inadequate criteria for diagnosing DID, basically limited to patients having distinct dissociative identities with their own memories, preferences and behavioral patterns, and episodes of amnesia (American Psychiatric Association, 2013; World Health Organization, 1993). Clinicians without experience of DID may therefore expect patients to present disruptions of identity during a consultation and spontaneously report memory problems. However, trauma specialists view DID as a ‘disorder of hiddenness’ because patients often find their dissociative symptoms bizarre and confusing and do not disclose them readily due to their shame and the phobia of inner experiences (Steele et al., 2005, 2016; Van der Hart et al., 2006). Instead, they tend to undermine their significance, hide them and not report them during consultations unless asked about them directly. Dissociative patients can also be unaware of their amnesia and ignore evidence for having done things they cannot remember because realizing that is too upsetting. Contrary to that, this study and the one conducted in 1999 in the Netherlands by Draijer and Boon, show that some people with personality disorders enthusiastically report DID symptoms by the book, and use the notion of multiple personalities to justify problems with emotional regulation, inner conflicts, or to seek attention. As with Dutch patients, Polish participants were preoccupied with their alternate personalities and two tried to present a ‘switch’ between parts. Their presentations were naïve and often mixed with lay information on DID. However, what they reported could be misleading for clinicians inexperienced in the dissociation field or those lacking the appropriate tools to distinguish a genuine dissociative disorder from an imitated one.

Therefore, understanding the subtleties about DID clinical presentation, especially those which are not thoroughly described in psychiatric manuals, is important to come up with a correct diagnosis and treatment plan. Various clinicians stress the importance of understanding the quality of symptoms and the mechanisms behind them in order to distinguish on the phenomenological level between borderline and DID patients (Boon and Draijer, 1993; Laddis et al., 2017). Participants in this study reported problems with identity, affect regulation and internal conflicts about expressing their impulses. Some of them also had somatic complaints. These symptoms are common in personality disorders and also in dissociative disorders, which are polysymptomatic by nature. However, the quality of these symptoms and psychological mechanisms behind them may be different. For a differential diagnosis, clinicians need to become familiar with the unique internal dynamics in people who have developed a structural dissociation of personality as a result of trauma. These patients try to cope with everyday life and avoid actively thinking about and discussing traumatic memories, or experiencing symptoms associated with them. Because of that avoidance, they find it challenging to talk about dissociative symptoms with a clinician. Besides experiencing fear of being labeled as insane and sent to hospital, there may be internal conflicts associated with disclosing information. For example, dissociative parts may forbid them to talk about symptoms or past experiences. This conflict can sometimes be indicated by facial expression, involuntary movements, spasms, and also felt by the clinician in his or her countertransference. In other words, it is not only *what* patients say about their experiences, but *how* they do this. Therapists’ observations and countertransference may help in assessing the quality of avoidance: How openly or easily do patients report symptoms or adverse life experiences? Is that associated with strong depersonalisation (detachment from feelings and sensations, being absent)? Is there evidence for internal conflicts, shame, fear or feeling blocked when talking about symptoms (often observed in facial expression, tone of voice)? Participants in this study were eager to talk about how others mistreated them and wanted to have that documented on paper. Difficult experiences in the past sometimes triggered intense emotions in them (anger, resentment, and deep sadness) but they did not avoid exploring and communicating these states. On the contrary, they eagerly shared an elaborate narrative of their sorrows and about their inner characters – the multiple personalities they were convinced they had. They became keen on DID and used a variety of resources to familiarize themselves with core symptoms. They also spontaneously reported them, as if they wanted to provide sound evidence about having DID and were ready to defend their diagnosis. Some planned their future based on it (an academic career, writing a book, or a film). During the interviews, it became clear that some perceived having an exotic diagnosis as an opportunity for seeking attention and feeling unique, exhibiting the drama of an ‘unseen child’ (see section “Theme 4”).

Understanding a few of the symptoms identified in this study can be useful for differential diagnosis: intrusions, voices, switches, amnesia, use of language, depersonalisation. How they are presented by patients and interpreted by clinicians is important.

### Intrusions

Triggered by external or internal factors (memories or anything associated with trauma) dissociative patients tend to relive traumatic experiences. In other words, they have intrusive memories, emotions or sensorimotor sensations contained by dissociative parts which are stuck in trauma. In addition to avoidance, this is another characteristic PTSD feature observed in the clinical presentation of DID patients (Van der Hart et al., 2010). Interestingly, participants in this study showed no evidence for intrusions (images, emotions or somatosensory experiences directly related to trauma), but rather problems with emotional regulation (illustrated in sections “Themes 1 and 2”). Asked about intrusive images, emotions or thoughts, some gave examples of distressing thoughts attacking self-image and blaming for their behavior. This, however, was related to attachment problems and difficulties with self-soothing. They also revealed a tendency to indulge themselves in these auto-critical thoughts instead of actively avoiding them, which is often a case in dissociative patients. Some intrusions reported by DID patients are somatoform in nature and connected with dissociative parts stuck in trauma time (Pietkiewicz et al., 2018). Although three participants in this study had very high scores in SDQ-20 indicating that they may have a dissociative disorder (scores of 50–60 are common in DID), further interviews revealed that they aggravated their symptoms and, in fact, had low levels of somatoform dissociation. This shows that tests results should be interpreted with caution and clinicians should always ask patients for specific examples of the symptoms they report.

### Voices

It is common for DID patients to experience auditory hallucinations (Dorahy et al., 2009; Longden et al., 2019). The voices usually belong to dissociative parts and comment on actions, express needs, likes and dislikes, and encourage self-mutilation. Subsequently, there may be conflicts between ‘voices,’ and the relationship with them is quite complex. Dorahy et al., 2009 observe that auditory hallucinations are more common in DID than in schizophrenia. In dissociative patients they are more complex and responsive, and already appear in childhood. Specifically, child voices are also to be expected in DID (97% in comparison to 6% in psychosis). None of our participants reported auditory hallucinations although one (Dominique) said she had imaginary friends from childhood. While this could sound like a dissociative experience, exploring their experiences showed she had a tendency to absorb herself in her fantasy world and vividly imagine characters in her story (see section “Theme 2”).

### Switches

Literature also shows that it is uncommon for avoidant dissociative patients to present autonomous dissociative parts to a therapist before a good relationship has been established and the phobia for inner experiences reduced (Steele et al., 2005). Sudden switches between dissociative personalities may occur only when the patient is triggered and cannot exercise enough control to hide his or her symptoms. Two participants in this study (Dominique and Karina) tried to present ‘alternate personalities’ and they actually announced this would happen, so that the interviewer did not miss them. Later on, they could relate to what happened during the alleged switch (no amnesia), maintaining the first-person perspective (I was saying/doing). Contrary to that, dissociative patients experience much shame and fear of disclosing their internal parts (Draijer and Boon, 1999). If they become aware that switches had occurred, they try to make reasonable explanations for the intrusions of parts and unusual behavior (e.g., I must have been very tired and affected by the new medicine I am taking).

### Amnesia

Dell (2006) mentions various indicators of amnesia in patients with DID. However, losing memory for unpleasant experiences may occur in different disorders, usually for behaviors evoking shame or guilt, or for actions under extreme stress (Laddis et al., 2017). All patients in this study had problems with emotional regulation and some said they could not remember what they said or did when they became very upset. With some priming, they could recall and describe events. For this reason, it is recommended to explore evidence for amnesia for pleasant or neutral activities (e.g., doing shopping or cleaning, socializing). According to Laddis et al. (2017) there are different mechanisms underlying memory problems in personality and dissociative disorders.

### Use of Language

Participants in this study often used clinical jargon (e.g., flashbacks, switches, and feeling depersonalized) which indicates they had read about dissociative psychopathology or received psycho-education. However, they often had lay understanding of clinical terms. A good example in this study was having ‘flashbacks’ of neutral or pleasant situations which had once been forgotten. Examples of nightmares did not necessarily indicate reliving traumatic events during sleep (as in PTSD) but expressed conflicts and agitation through symbolic, unrealistic, sometimes upsetting dreams. When talking about behavior of other parts and their preferences, they often maintained a first-person perspective. Requesting patients to provide specific examples is thus crucial.

### Depersonalisation

Detachment from feelings and emotions, bodily sensations and external reality is often present in various disorders (Simeon and Abugel, 2006). While these phenomena have been commonly associated with dissociation, Holmes et al. (2005) stress the differences between detachment (which can be experienced by both dissociative and non-dissociative patients) and compartmentalisation, associated with the existence of dissociative parts. Allen et al. (1999) also stress that extreme absorptive detachment can interfere with noticing feelings and bodily sensations, and also memory. Some participants in this study tended to enter trance-like states or get absorbed in their inner reality, subsequently getting detached from bodily sensations. They also described their feeling of emptiness in terms of detachment from feelings. Nevertheless, none of them disclosed evidence for having distinct dissociative parts. Some of their statements might have been misleading; for example, when they attributed anger attacks to other parts, not-me (see: Dominique in section “Theme 2”). One might suspect it could be evidence for autonomous dissociative parts. However, these participants seem to have had unintegrated, unaccepted self-states and used the concept of DID to make meaning of their internal conflicts. In their narrative they maintained the first-person narrative. None of them provided sound evidence for extreme forms of depersonalisation, such as not feeling the body altogether or out-of-body experiences.

There can be many reasons why people develop symptoms which resemble those typical of DID. Suggestions about a dissociative disorder made by healthcare providers can help people justify and explain inner conflicts or interpersonal problems. In this study several clinicians had suggested a dissociative disorder or DID to the patient. Literature on multiple personalities and therapy focused on them, and using expressions such as ‘parts’, ‘dissociating’, ‘switches,’ can also encourage demonstrating such symptoms. There are also secondary gains explained in this study, such as receiving attention and care. Draijer and Boon (1999) observe that people with borderline features justified shameful behavior and avoided responsibility by attributing their actions to ‘alter personalities.’ Such people can declare amnesia for their outbursts of anger, or hitting partners. Others explained their identity confusion and extreme emptiness using the DID model. All their participants reported emotional neglect and felt unseen in their childhood, so they adopted a new DID-patient identity to fill up inner emptiness (Draijer and Boon, 1999). Just like the participants in this study, they were angry when that diagnosis was disconfirmed during the assessment, as if the clinician had taken away something precious from them. This shows that communicating the results should be done with understanding, empathy and care. Patients and clinicians need to understand and discuss reasons for developing a DID-patient identity, its advantages and pitfalls.

In countries where clinicians are less familiar with the dissociative pathology, there may be a greater risk for both false-negative and false-positive DID diagnoses. The latter is caused by the growing popularity of that disorder in media and social networks. People who try to make meaning of their emotional conflicts, attachment problems and difficulties in establishing satisfactory relationships, may find the DID concept attractive. It is important that clinicians who rule out or disconfirm DID, also provide patients with friendly feedback that encourages using treatment for their actual problems. Nevertheless, this may still evoke strong reactions in patients whose feelings and needs have been neglected, rejected or invalidated by significant others. Disconfirming DID may be experienced by them as an attack, taking something away from them, or an indication that they lie.

### Limitations and Further Directions

Among the 85 people who participated in a thorough diagnostic assessment, there were six false-positive DID cases, and this study focused on their personal experiences and meaning attributed to the diagnosis. Because IPA studies are highly idiographic, they are by nature limited to a small number of participants. There were two important limitations in this research. Firstly, information about the level of psychoform symptoms has not been given, because the validation of the Polish instrument used for that purpose is not complete. Secondly, TADS-I used for collecting clinical data about trauma-related symptoms and dissociation has not been validated, either. Because there are no gold standards in Poland for diagnosing dissociative disorders, video-recordings of diagnostic interviews were carefully analyzed and discussed by all authors to agree upon the diagnosis. Taking this into consideration, further qualitative and quantitative research is recommended to formulate and validate more specific diagnostic criteria for DID and guidelines for the differential diagnosis.

## Conclusion

Clinicians need to understand the complexity of DID symptoms and psychological mechanisms responsible for them in order to differentiate between genuine and imitated post-traumatic conditions. There are several features identified in this study which may indicate false-positive or imitated DID shown in Table 4, which should be taken into consideration during diagnostic assessment. In Poland, as in many countries, this requires more systematic training in diagnosis for psychiatrists and clinical psychologists in order to prevent under- and over-diagnosis of dissociative disorders, DID in particular. It is not uncommon that patients exaggerate on self-report questionnaires when they are invested in certain symptoms. In this study, all participants had scores above the cut-off score of 28 on the SDQ-20, a measure to assess somatoform dissociation, which suggested it was probable they had a dissociative disorder. However, during a clinical diagnostic interview they did not report a cluster of somatoform or psychoform dissociative symptoms and did not meet criteria for any dissociative disorder diagnosis. Clinicians also need to go beyond the face value of a patient’s responses, ask for specific examples, and notice one’s own countertransference. Draijer and Boon (1999) observed that DID patients were often experienced by clinicians as very fragile, and exploring symptoms with people with personality disorders (who try to aggravate them and control the interview) can evoke tiredness or even irritability. It is important that clinicians understand their own responses and use them in the diagnostic process.

**TABLE 4:** Red flags for identifying false-positive or imitated DID.

This table enumerates suggestive features of false positive or imitated DID cases identified in this study, which should be taken into consideration during diagnostic assessment.
1. Directly or indirectly expects to confirm self-diagnosed DID.
2. DID previously suggested by someone (friend, psychologist, and doctor) without thorough clinical assessment.
3. Keen on DID diagnosis and familiarized with symptoms: read books, watched videos, talked to other patients, participated in a support group for dissociative patients.
4. Uses clinical jargon: parts, alters, dissociating, switch, depersonalisation, etc.
5. Reveals little avoidance: eagerly talks about painful experiences and dissociation, no indicators for genuine shame or inner conflicts associated with disclosing symptoms or parts.
6. Readily justifies losing control of emotions and unacceptable or shameful behavior in terms of not being oneself or being influenced by an alternative personality.
7. No evidence for the intrusions of unwanted and avoided traumatic memories or re-experiencing them in the present.
8. Denies having ego-dystonic thoughts or voices, especially starting in early childhood and child-like voices. Note: Dissociative patients may be afraid, ashamed, or feel it is forbidden to talk about the voices.
9. No evidence of amnesia for neutral or pleasant everyday activities, e.g., working, doing shopping, socializing, playing with children.
10. Tries to control the interview and provide evidence for having DID, e.g., eagerly reports dissociative symptoms without being asked about them.
11. Announces and performs a switch between personalities during clinical assessment, especially before a good relationship with the clinician and trust has been established.
12. Finds apparent gains associated with having DID: receives special interest from family and friends with whom symptoms and personalities are eagerly discussed, runs support groups, blogs or video channels for people with dissociative disorders.
13. Gets upset or disappointed when DID is not confirmed, e.g., demands re-evaluation, excuses oneself for not being accurate enough in giving right answers, wants to provide more evidence.

While psycho-education is considered a crucial element in the initial treatment of dissociative disorders (Van der Hart et al., 2006; Howell, 2011; Steele et al., 2016), patients whose diagnosis has not been confirmed by a thorough diagnostic assessment should not be encouraged to develop knowledge about DID symptomatology, because this may affect their clinical presentation and how they make meaning of their problems. Subsequently, this may lead to a wrong diagnosis and treatment, which can become iatrogenic.

## Data Availability Statement

The datasets generated for this study are not readily available because data contain highly sensitive clinical material, including medical data which cannot be shared according to local regulations. Requests to access the datasets should be directed to IP, ipietkiewicz@swps.edu.pl.

## Ethics Statement

The studies involving human participants were reviewed and approved by Ethical Review Board at the SWPS University of Social Sciences and Humanities. The patients/participants provided their written informed consent to participate in this study.

## Author Contributions

IP collected qualitative data, performed the analysis, and prepared the manuscript. AB-N transcribed and analyzed the interviews and helped in literature review and manuscript preparation. RT performed psychiatric assessment and helped in data analysis and manuscript preparation. SB helped in data analysis and manuscript preparation. All authors contributed to the article and approved the submitted version.

## Conflict of Interest

The authors declare that the research was conducted in the absence of any commercial or financial relationships that could be construed as a potential conflict of interest.
